# Organoaxial gastric volvulus secondary to hiatal hernia successfully treated with laparoscopic surgery: a case report

**DOI:** 10.1093/jscr/rjag508

**Published:** 2026-06-25

**Authors:** Ahmet Salhat, Caner Akgül, Yacob Ledamo Habeş

**Affiliations:** Department of General Surgery, Gebze Fatih State Hospital, Osman Yılmaz Neighborhood, İstanbul Street No: 62, Gebze, Kocaeli 41400, Türkiye; Department of General Surgery, Medical Park Kocaeli Hospital, Ovacık Mahallesi, D100 Karayolu Caddesi No: 34, İzmit, Kocaeli 41090, Türkiye; Department of General Surgery, Gebze Fatih State Hospital, Osman Yılmaz Neighborhood, İstanbul Street No: 62, Gebze, Kocaeli 41400, Türkiye

**Keywords:** gastric volvulus, organoaxial volvulus, hiatal hernia, laparoscopic surgery, Nissen fundoplication

## Abstract

Gastric volvulus is a rare but potentially life-threatening condition characterized by abnormal rotation of the stomach. Early diagnosis and surgical management are essential to prevent ischemia and necrosis. We report the case of a 56-year-old woman presenting with abdominal pain and vomiting. Contrast-enhanced computed tomography demonstrated a markedly distended stomach with abnormal orientation consistent with organoaxial gastric volvulus. After initial decompression with a nasogastric tube, the patient underwent laparoscopic exploration. Intraoperatively, the proximal stomach was found herniated through the hiatus, causing organoaxial gastric volvulus. The stomach was reduced from the thorax, detorsion was performed, and hiatal repair with cruroplasty and Nissen fundoplication was completed. No ischemia or necrosis was observed. The patient had an uneventful postoperative course and was discharged on postoperative day four. This case reinforces the role of early diagnosis and laparoscopic management in achieving favorable outcomes in gastric volvulus associated with hiatal hernia.

## Introduction

Gastric volvulus is an uncommon clinical condition defined as abnormal rotation of the stomach by more than 180°, which may result in obstruction, ischemia, and necrosis [1, 2]. The condition is classified into organoaxial and mesenteroaxial types based on the axis of rotation [1]. Organoaxial volvulus is the most common form and is frequently associated with diaphragmatic defects such as hiatal hernia [1, 3, 4]. Early diagnosis and timely surgical intervention are critical to prevent life-threatening complications [2, 5]. Although rare, gastric volvulus carries a reported mortality rate of up to 30%–50% in untreated acute cases due to gastric ischemia and perforation [1, 5].

## Case presentation

A 56-year-old woman with a history of hypertension presented to the emergency department with complaints of abdominal pain and vomiting. On physical examination, mild abdominal tenderness and distension were present without signs of defense or rebound tenderness. Laboratory tests revealed leukocytosis with a white blood cell count of 13 600/mm^3^, while other laboratory parameters were within normal limits.

Contrast-enhanced abdominal computed tomography (CT) demonstrated a distended stomach with displacement of the greater and lesser curvatures. The antropyloric region and part of the duodenum were located in the right upper quadrant of the abdomen. These findings were consistent with organoaxial gastric volvulus (Fig. 1).

**Figure 1:**
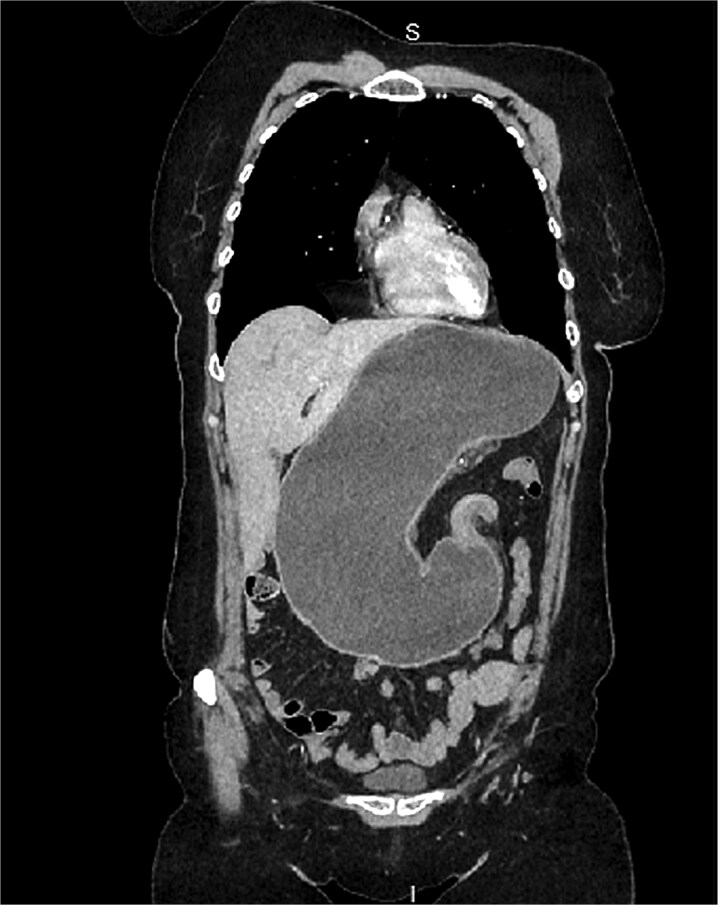
Contrast-enhanced CT demonstrated a markedly distended stomach with abnormal orientation consistent with organoaxial gastric volvulus.

A nasogastric tube was inserted for gastric decompression, yielding approximately 1600 ml of bilious fluid. The patient was admitted for further evaluation and monitoring. Upper gastrointestinal endoscopy performed the following day revealed findings consistent with gastric volvulus along with mucosal edema and patchy gastric ulcers (Fig. 2).

**Figure 2:**
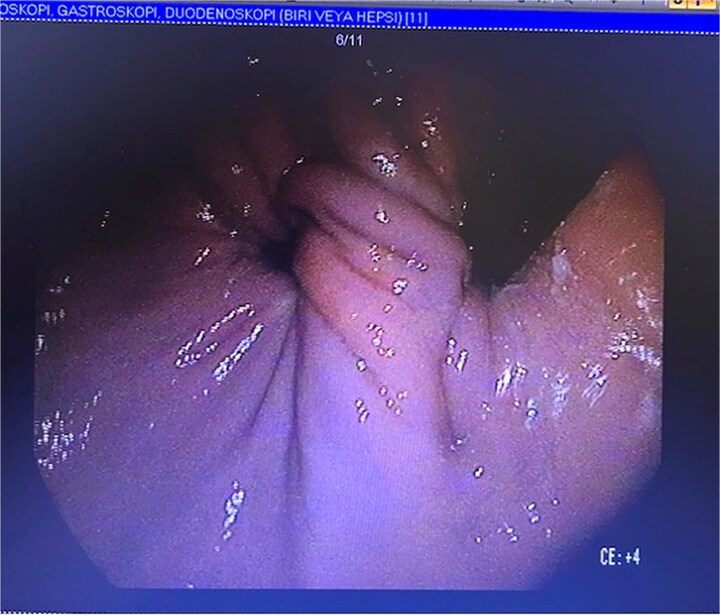
Endoscopic view demonstrating twisted gastric folds and distorted lumen consistent with gastric volvulus.

Emergency surgical intervention was planned. Laparoscopic exploration revealed that the proximal portion of the stomach had herniated through the esophageal hiatus, resulting in organoaxial gastric volvulus (Fig. 3). The stomach was reduced into the abdominal cavity and detorsion was performed (Fig. 4). No evidence of gastric ischemia or necrosis was observed. Hiatal repair with cruroplasty and Nissen fundoplication was performed (Figs 5 and 6). The postoperative course was uneventful, and the patient was discharged on postoperative day four in good clinical condition.

**Figure 3:**
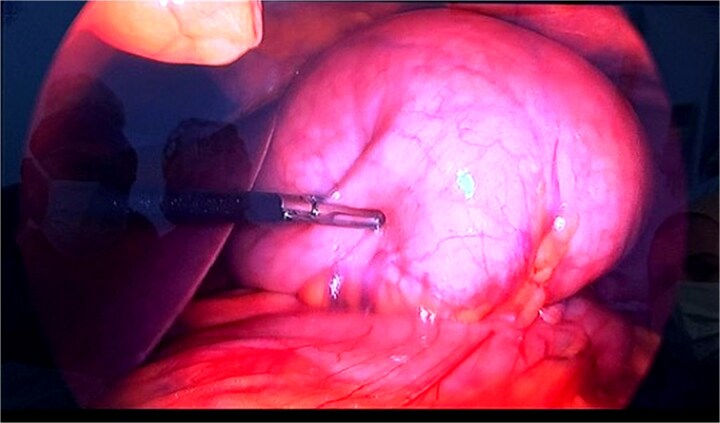
Laparoscopic view of organoaxial gastric volvulus.

**Figure 4:**
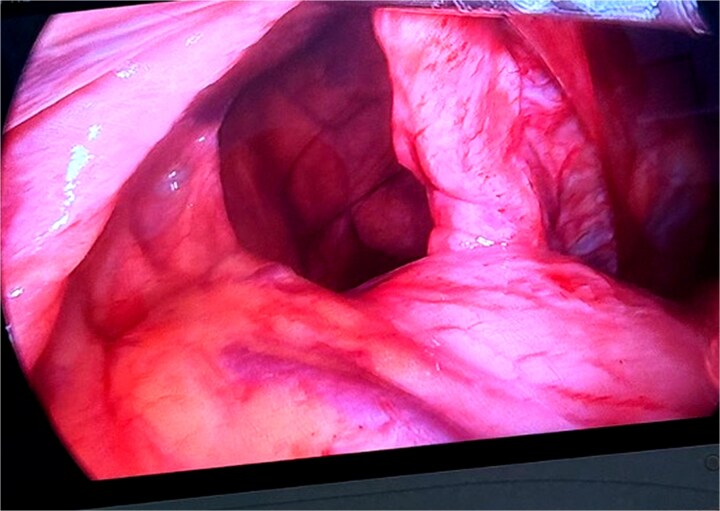
Intraoperative image showing the hiatal defect and herniation of the stomach into the thoracic cavity.

**Figure 5:**
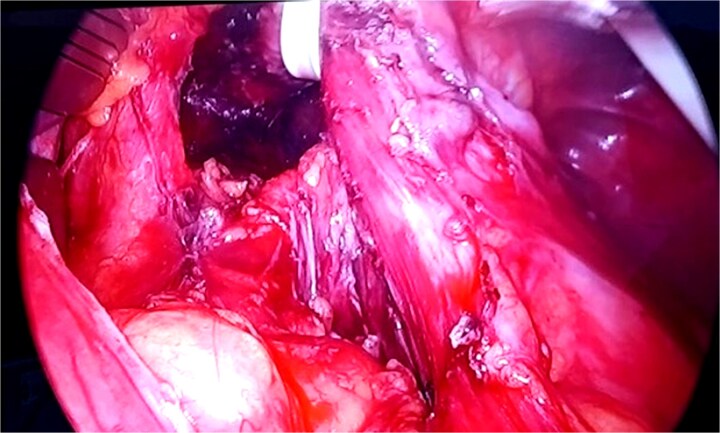
Dissection of the hiatus and preparation for hiatal repair.

**Figure 6:**
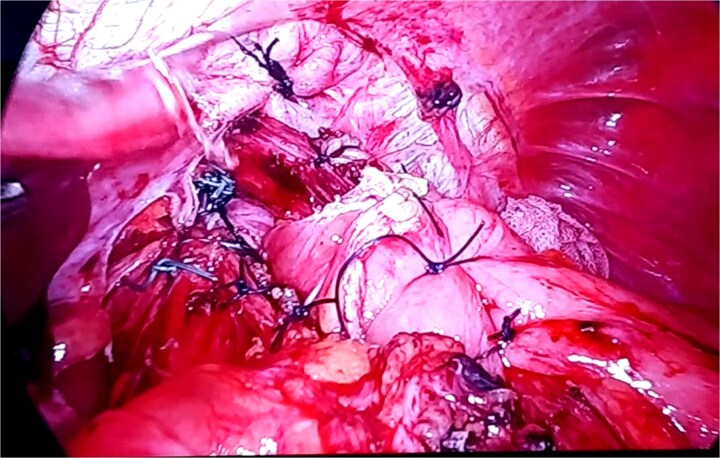
Completed cruroplasty and Nissen fundoplication after reduction of the volvulus.

## Discussion

Gastric volvulus is a rare but potentially fatal condition if not promptly diagnosed and treated [1, 2]. Organoaxial volvulus accounts for approximately two-thirds of cases and often occurs in association with diaphragmatic defects such as hiatal hernia [1, 3]. Clinical presentation typically includes abdominal pain, vomiting, and gastric distension [2, 6].

CT plays a crucial role in the diagnosis by demonstrating abnormal gastric rotation and anatomical displacement [7]. Early decompression with a nasogastric tube may temporarily relieve symptoms; however, definitive treatment is surgical [2, 5].

Laparoscopic surgery has become the preferred approach in stable patients, allowing reduction of the volvulus, repair of the underlying defect, and prevention of recurrence [8, 9]. Procedures such as cruroplasty and fundoplication are frequently performed when hiatal hernia is present [3, 8].

Similar cases of organoaxial gastric volvulus associated with hiatal hernia have been reported in the literature, most of which were managed surgically with favorable outcomes. Previous studies have emphasized the importance of early diagnosis and prompt surgical intervention to prevent ischemia and necrosis [1, 3, 8, 9]. In line with the literature, our patient was successfully treated with laparoscopic reduction and hiatal repair, resulting in an uneventful recovery. Compared to previously reported cases, our case highlights the effectiveness of minimally invasive surgery and supports its role as the preferred treatment approach in stable patients.

## Conclusion

Organoaxial gastric volvulus associated with hiatal hernia is a rare but serious condition that requires prompt diagnosis and treatment. Laparoscopic reduction combined with hiatal repair and fundoplication is a safe and effective treatment option with favorable outcomes.
